# Acute Eosinophilic Myocarditis and Heart Failure As the First Manifestation of Eosinophilic Granulomatosis With Polyangiitis (EGPA) in an Asthmatic Patient: A Diagnostic Challenge

**DOI:** 10.7759/cureus.98175

**Published:** 2025-11-30

**Authors:** Ei M Mon, Trisha Singh, Laith Aldabbagh, Aung Hein, Christopher J Boos

**Affiliations:** 1 Department of Cardiology, University Hospitals Dorset NHS Foundation Trust, Bournemouth, GBR; 2 Department of Cardiology, Poole Hospital, University Hospitals Dorset NHS Foundation Trust, Poole, GBR; 3 Department of Postgraduate Medicine, Bournemouth University, Bournemouth, GBR

**Keywords:** acute kidney injury, asthma, eosinophilic granulomatosis with polyangiitis (egpa), heart failure, myocarditis, vasculitis

## Abstract

Eosinophilic granulomatosis with polyangiitis (EGPA) is a rare necrotizing vasculitis that affects multiple organ systems. Asthma is a hallmark clinical feature of the syndrome. Cardiac involvement is uncommon but constitutes one of the most severe and potentially life-threatening manifestations of the disease. We present a challenging diagnostic case of a 68-year-old woman with a history of asthma who presented with features suggestive of acute coronary syndrome and elevated cardiac troponin levels. Coronary angiography revealed only mild atheroma. The combination of a negative angiogram, marked eosinophilia, significantly elevated troponin, and cardiac dysfunction on imaging led to the diagnosis of EGPA-associated myocarditis. In this case, the disease initially manifested with cardiac symptoms, followed by subsequent renal impairment. Corticosteroid and immunosuppressive therapy effectively and rapidly controls disease activity. A multidisciplinary approach is essential in achieving optimal patient outcomes.

## Introduction

Eosinophilic granulomatosis with polyangiitis (EGPA), formerly known as Churg-Strauss syndrome (CSS), is a rare systemic autoimmune vasculitis predominantly involving small- to medium-sized vessels with an estimated global incidence of fewer than 2.5 cases per 100,000 adults annually [1]. The disease onset usually occurs between the third and fourth decades, affecting both sexes equally [1]. It can affect multiple organs, with cardiac involvement representing the leading cause of morbidity and mortality [2]. 

Clinically significant cardiac involvement occurs in 27-47% of cases, with myocarditis being the most frequent manifestation [3]. Other cardiac presentations include pericarditis, pericardial effusion, acute myocardial infarction, severe heart failure, cardiogenic shock, and death [3].

Eosinophilic myocarditis (EM) is an infrequent inflammatory myocardial condition characterized by eosinophil infiltration of the myocardial tissue, often associated with peripheral eosinophilia. Although eosinophilic myocarditis has been documented in association with drug-related hypersensitivity reactions, immune-mediated diseases such as EGPA, various forms of hypereosinophilic syndrome (HES), myeloproliferative disorders, viral and parasitic infections, as well as malignant conditions, the etiology remains idiopathic in a significant proportion of cases [4].

We report a rare case of a 68-year-old asthmatic patient who initially presented with symptoms mimicking acute coronary syndrome and was later diagnosed with EGPA-associated myocarditis, requiring high-dose corticosteroids and immunosuppressive therapy. This case highlights the diagnostic challenges of EGPA presenting as acute myocarditis and emphasizes the importance of early recognition and timely treatment.

## Case presentation

A 68-year-old female patient presented with central chest tightness that began upon waking. She described the pain as a constant heavy pressure on her chest. It radiated to her jaw and was associated with a pins-and-needles sensation in her left arm. During the three weeks prior to admission, she has experienced intermittent episodes of left-sided chest discomfort along with progressive shortness of breath on minimal exertion. She has a history of asthma, diagnosed during her teenage years. Her asthma has been generally well controlled with regular use of a Fostair and Salamol inhaler. However, her asthma control deteriorated since a recent trip to Canada, and she experienced recurrent exacerbations and chest infections requiring repeated courses of oral prednisolone and antibiotics. She also had a history of recurrent sinusitis, but no known nasal polyps. 

**Figure 1:**
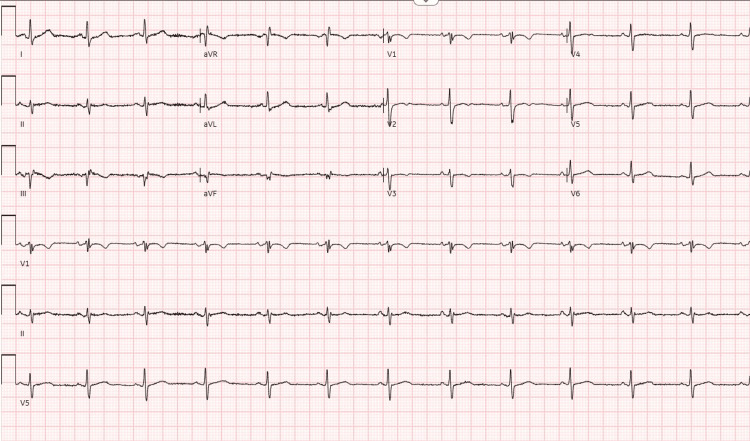
ECG at first admission showing anterior T wave inversion in V1-V3

Cardiovascular examination was unremarkable, and lung auscultation was clear. Her vital signs were stable, with a blood pressure of 118/86 mmHg, pulse rate of 105 beats per minute, and oxygen saturation of 97% on room air. Her 12-lead ECG showed sinus rhythm with anterior T-wave inversions in leads V1-V3 (Figure 1). High-sensitivity troponin (Hs-cTnT) levels were elevated, rising from 235 to 249 ng/L (reference, 0-13ng/L). Chest X-ray (CXR) showed no acute abnormalities. Transthoracic echocardiogram (TTE) demonstrated preserved left ventricular systolic function with no pericardial effusion. Blood tests on admission revealed an elevated eosinophil count of 2.7 ×10⁹/L (reference, 0-0.5×10⁹/L), in contrast to a normal value two months previously. There was a mildly increased white cell count of 11.9 x10⁹/L (reference, 4-10 x10⁹/L), serum C-reactive protein of 22mg/L (reference, 0-9mg/L) and NT-proBNP of 421 ng/L (reference, <400ng/l), with normal renal, thyroid and liver function.

The patient was managed as a non-ST elevation myocardial infarction (NSTEMI) and received loading doses of oral aspirin 300 mg and clopidogrel 300 mg, along with fondaparinux 2.5 mg subcutaneously. Due to ongoing intermittent chest pain, she was transferred to the coronary care unit (CCU) for initiation of a glyceryl trinitrate (GTN) infusion. Her pain resolved with GTN infusion and morphine. 

The following day, an invasive coronary angiogram was performed, revealing minor atheroma in the proximal left anterior descending artery (LAD) only. A CT pulmonary angiogram (CTPA) was performed, which excluded pulmonary embolism and other significant lung pathology. She was discharged on dual antiplatelet therapy, and an urgent outpatient cardiac MRI (CMR) was arranged to further evaluate the possibility of myopericarditis.

The patient re-presented eight days post discharge with ongoing central chest pain, exacerbated by lying flat and relieved by sitting forward, accompanied by dyspnoea. Cardiovascular examination revealed normal heart sounds with no elevation of jugular venous pressure (JVP), although mild bilateral ankle pitting oedema was present. Respiratory examination demonstrated a mild expiratory wheeze, and she required 2L of oxygen.

**Figure 2:**
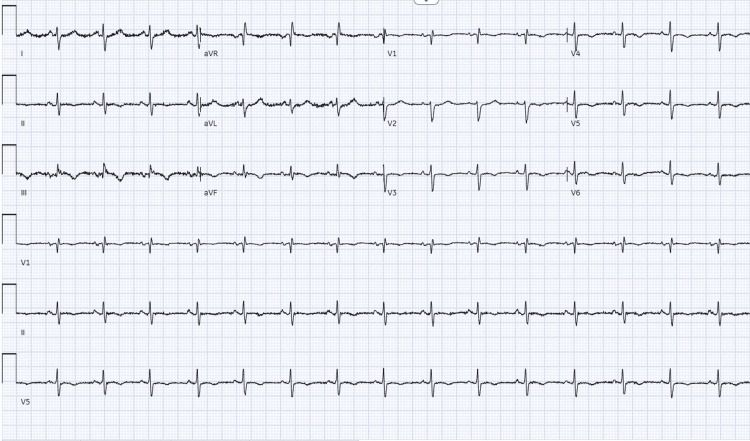
ECG at second admission showing new T wave inversion in V4-V6

**Figure 3:**
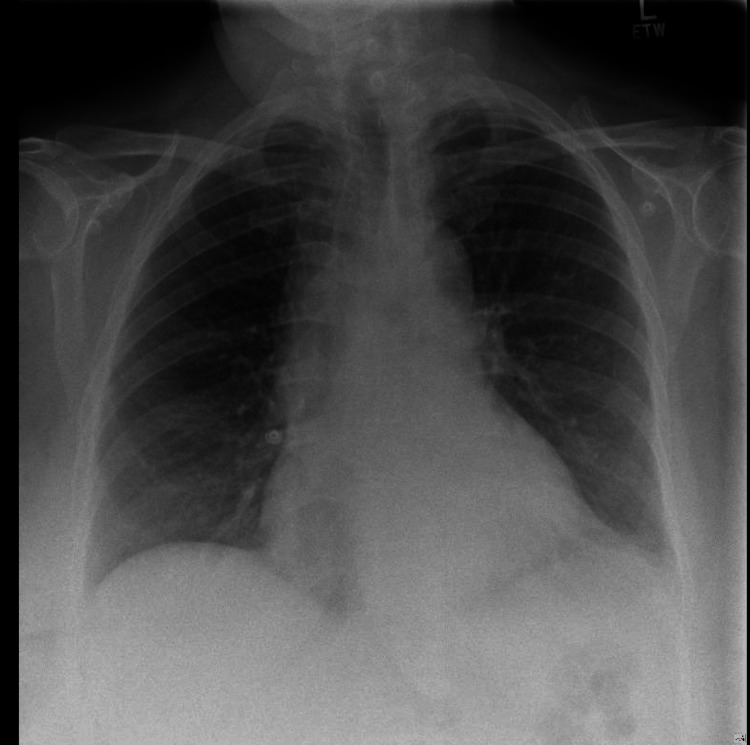
Chest X-ray showing left lower zone airspace opacification with small left pleural effusion

Her repeat ECG again showed sinus rhythm but with new T wave inversion in V4-V6 (Figure 2). Hs-cTnT levels were significantly elevated, rising from 863 to 911 ng/L, representing a further increase compared with the previous admission. Her repeat CXR demonstrated left lower zone airspace opacification, consistent with infection, along with a small left pleural effusion (Figure 3). Her admission blood eosinophil count was markedly elevated at 7.5 ×10⁹/L, compared with 3.2 ×10⁹/L at her previous discharge. Eosinophilia and left-shifted neutrophils were seen on the blood film. Blood cultures were sterile, and viral screening for COVID-19, influenza A/B, and respiratory syncytial virus (RSV) was negative. 

The patient was managed for myopericarditis with oral colchicine 500 µg twice daily, oral ibuprofen 400 mg three times daily, with 30 mg once daily of oral lansoprazole, and her aspirin and clopidogrel were discontinued. Concurrently, she received salbutamol nebulisers and oral prednisolone 40 mg for asthma exacerbation, as well as oral doxycycline 200 mg stat, followed by 100 mg once daily for a chest infection. She was reviewed by the respiratory team, who recommended a series of investigations, including immunological tests and connective tissue disease screening. The results of the key immunological tests are shown in Table 1. 

**Table 1:** Laboratory test results at second admission RAST: radioallergosorbent test; VCA: Viral Capsid Antigen; ELISA: enzyme-linked immunosorbent assay

Tests	Patient Value	Reference Range
*Aspergillus fumigatus* Ig G	26.00 mgA/L	0-40
Total Ig E	232.00 kU/L	
*Aspergillus fumigat *RAST	0.36 kUA/L	0.0-0.34
Myeloperoxidase antibody level (MPO)	0.40 IU/mL	0- 3.49
Proteinase 3 antibody level ( PR3)	< 0.60 IU/mL	0-1.99
Connective tissue disease screen	0.2	0.0-0.6
Anti-streptolysin O titre	< 200	
Cytomegalovirus IgG antibody	Not detected	
Cytomegalovirus Ig M Antibody	Not detected	
Epstein Barr VCA IgG Antibody	Detected	
Epstein Barr VCA IgM Antibody	Not detected	
Syphilis Antibody	Not detected	
Strongloides ELISA	Negative	

On the eighth day of her second admission, the patient developed atrial fibrillation with a rapid ventricular response. Rate control was achieved with oral digoxin 125 µg once daily, and she was initiated on oral apixaban at a dose of 5 mg twice daily.

**Figure 4:**
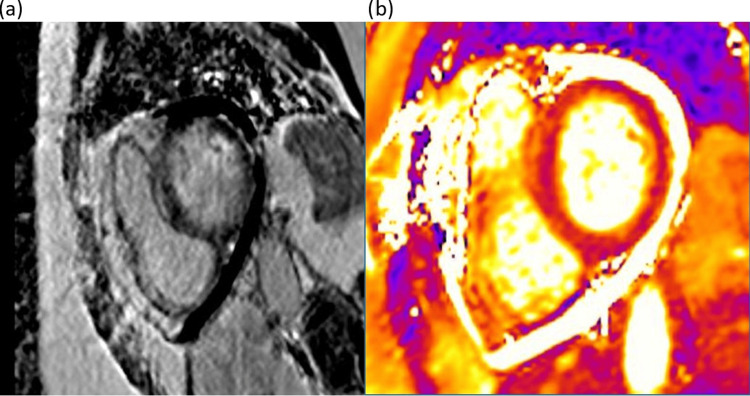
Short axis image of left ventricle demonstrating patchy mid wall LGE (a) and elevated T2 values (b) LGE: late gadolinium enhancement

A repeat echocardiogram demonstrated severe biventricular systolic dysfunction with an estimated left ventricular ejection fraction of 20-25%. A small, loculated pericardial effusion (<1 cm) was noted around the right ventricular free wall (Videos 1, 2). During echocardiography, the patient was in rapid atrial fibrillation, and a suboptimal acoustic window limited the accurate quantification of the ejection fraction; however, there was clear evidence of left ventricular systolic dysfunction. Her CMRI (on day four of admission) demonstrated diffuse myocardial oedema throughout the entire myocardium, accompanied by diffuse, patchy mid-wall late gadolinium enhancement consistent with acute myocarditis (Figure 4). A very small pericardial effusion was noted, without evidence of pericardial thickening. Left ventricular systolic function was globally mildly impaired, with an ejection fraction of 50%. At this stage, the differential diagnoses include EGPA, invasive aspergillosis with myocarditis, and hypereosinophilic syndrome with myocardial involvement. 

**Video 1:** Transthoracic echocardiogram (PLAX view) PLAX: parasternal long axis view

**Video 2:** Transthoracic echocardiogram (subcostal view)

On day five of admission, the patient’s renal function began to deteriorate, progressing to acute kidney injury (AKI) stage III by the next day, with estimated glomerular filtration rate (eGFR) declining from 66 to 23 ml/minute/1.73 m^2^. Her Hs-cTnT rose progressively, reaching a peak value of 1,453 ng/L. She was subsequently reviewed by both the renal and rheumatology teams as an inpatient. A renal ultrasound demonstrated normal kidney sizes and appearance. An urgent T-Spot test was also arranged at the request of the rheumatology team. The results of the blood investigations are given in Table 2.

**Table 2:** Laboratory test results on Day 5 of second admission ESR: erythrocyte sedimentation rate

Tests	Patient Values	Reference Ranges
Serum glomerular basement membrane antibodies level	<1.5 U/mL	0-6.9
Complement-third component-C3	1.78 g/L	0.79-1.8
Complement- fourth component-C4	0.30 g/L	0.12-0.36
IgG	10.86g/L	6.0-16.0
IgA	4.33g/L	0.8-4.0
IgM	0.45g/L	0.5-2.0
Serum total protein	64g/L	60-80
Serum albumin	30g/L	32-46
Serum Electrophoresis	No paraprotein detected	
Free kappa	28.0mg/L	3.3-19.4
Free Lambda	23.0mg/L	5.7-26.3
Kappa: Lambda ratio	1.22	
ESR	24mm/h	1-20
T-SPOT Assay	Indeterminate	

**Figure 5:**
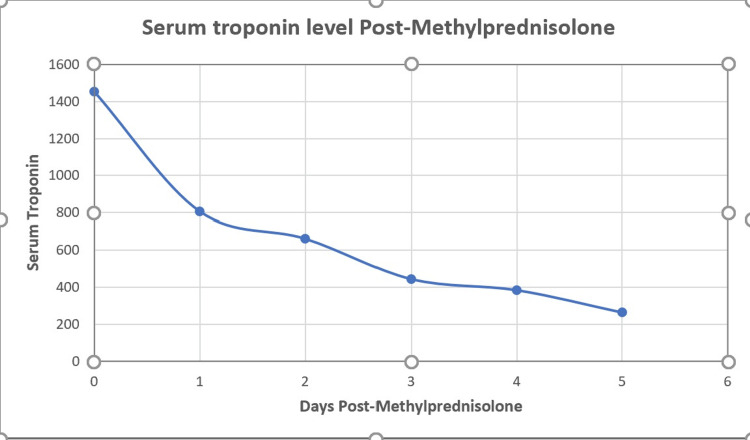
Progressive decline in serum troponin levels following initiation of intravenous methylprednisolone therapy

Following a multidisciplinary team (MDT) discussion involving cardiology, rheumatology, nephrology, and respiratory specialists, a diagnosis of EGPA was established based on the presence of asthma, acute myocarditis, marked peripheral eosinophilia, and acute renal injury. High-dose corticosteroid therapy was initiated with intravenous methylprednisolone 1 g daily for three consecutive days, followed by oral prednisolone 60 mg initially once daily with a tapering oral prednisolone regimen thereafter. In addition, intravenous cyclophosphamide was initiated at a dose of 125 mg/kg, alongside prophylactic co-trimoxazole 960 mg three times per week, in accordance with rheumatology recommendations. Following initiation of IV methylprednisolone, the patient showed a significant decrease in troponin levels (Figure 5), along with improvement in renal function.

A repeat echocardiogram five days after IV methylprednisolone demonstrated normal biventricular function. Once renal function improved, heart failure medications were initiated, including ramipril 1.25 mg once daily, dapagliflozin 10 mg once daily, and eplerenone 25 mg once daily. The patient completed six cycles of IV cyclophosphamide in the outpatient setting. She was followed up regularly in the rheumatology and cardiology clinics and remains clinically stable with sustained remission at four months of follow-up.

## Discussion

The diagnosis of EGPA is often challenging, as it can manifest with diverse clinical presentations due to its involvement of multiple organ systems, including the renal, gastrointestinal, neurological, dermatological, and cardiac systems. Myocardial involvement in EGPA can mimic acute coronary syndrome, often leading to diagnostic delay [1].

EGPA myocardial involvement typically progresses through three distinct phases. The eosinophilic infiltration and cardiac injury in the initial phase lead to the development of eosinophilic myocarditis. The second stage is the thrombotic phase, characterized by hypercoagulability and intracardiac thrombosis affecting the coronary arteries and ventricular chambers. The late fibrotic stage results in extensive scarring and the development of irreversible cardiac dysfunction [5]. Approximately 60% of patients with EGPA are seronegative for antineutrophil cytoplasmic antibodies (ANCA) [3]. Patients with EGPA-associated myocarditis often exhibit significant eosinophilia and are typically ANCA-negative, as observed in our case [1]. Studies indicate that 90% of EGPA cases complicated by myocarditis have underlying asthma [3]. Renal involvement in EGPA is relatively uncommon, occurring in approximately 25% of cases [6]. Consequently, the concurrent and clinically significant renal and cardiac involvement as observed in our patient represents a particularly rare presentation of the disease.

In our case, the diagnosis was established using the 2022 American College of Rheumatology (ACR)/European Alliance of Associations for Rheumatology (EULAR) classification criteria for EGPA, with the patient scoring 8 points based on the presence of obstructive airway disease and marked blood eosinophilia. A total score of ≥6 supports the classification of EGPA [7]; this confirms the diagnosis in our patient. The classification criteria are outlined in Table 3.

**Table 3:** ACR/EULAR 2022 classification criteria for eosinophilic granulomatosis with polyangiitis ACR: American College of Rheumatology; EULAR: European Alliance of Associations for Rheumatology Table Source: Noiri et al., 2025 [7]; under license CC BY 4.0, Attribution 4.0 International Deed

Clinical criteria	Scores
Obstructive airway disease	+3
Nasal polyps	+3
Mononeuritis multiplex	+1
Laboratory and biopsy criteria	
Blood eosinophil count ≥ 1×10⁹/L	+5
Extravascular eosinophilic-predominant inflammation on biopsy	+2
Positive cytoplasmic anti-neutrophil cytoplasmic antibody(c-ANCA) or anti- proteinase 3 (PR3) antibodies	-3
Haematuria	-1

TTE and CMR are valuable tools for detecting myocardial involvement in EGPA [1]. CMR can further characterize the diagnosis and assess the extent of myocardial injury and necrosis. Nevertheless, endomyocardial biopsy (EMB) is the gold standard for definitive diagnosis [8]. In our case, the combination of clinical features and immunological findings was sufficient to fulfil the diagnostic criteria. Due to the elevated thrombotic risk and necessity for ongoing anticoagulation, a myocardial biopsy was not pursued. Invasive coronary angiography, or, in selected cases, CT coronary angiography, is recommended to exclude underlying coronary artery disease [8]. In our patient, coronary angiography was performed during the initial admission due to symptoms mimicking coronary artery disease. 

For patients with organ- or life-threatening disease, prompt remission induction with glucocorticoids combined with either cyclophosphamide or rituximab is recommended to rapidly control disease activity and prevent irreversible organ damage [8]. Our patient responded well to intravenous methylprednisolone and cyclophosphamide, with significant improvement in her cardiac and renal function following treatment. 

## Conclusions

EGPA-associated myocarditis can initially present with symptoms mimicking an acute coronary syndrome, which may lead to misdiagnosis in the early stages. This condition should be included in the differential diagnosis of chest pain in patients with a history of asthma. In cases of myocarditis with an initially unclear etiology, monitoring eosinophil counts is essential, as rising levels may reveal an underlying cause and indicate ongoing disease activity, as illustrated in our patient. Although a marked decline in renal function is uncommon in patients with EGPA-related myocarditis, close daily monitoring remains essential, as deterioration can occur abruptly. Early involvement of a multidisciplinary team is crucial in the management of EGPA-associated myocarditis. Early initiation of high-dose methylprednisolone and immunosuppressive therapy can rapidly control disease activity and reduce the risk of long-term organ damage. 
